# Supplementary material for: Towards a High-Affinity Peptidomimetic Targeting Proliferating Cell Nuclear Antigen from Aspergillus fumigatus
Source: J Fungi (Basel). 2023 Nov 10;9(11):1098. doi: 10.3390/jof9111098 (PMC10672205; doi:10.3390/jof9111098)
Supplement: Supplementary file 1 [file jof-09-01098-s001.zip › jof-2683672-supplementary.pdf]

Electronic Supplementary Information

Table S1. p21 Peptide Surface Plasmon Resonance (SPR) data against AfumPCNA. The Top Conc is the highest concentration of 8x 1 in 2 dilutions used to calculate the steady state affinity. K_D is the affinity constant. SD, standard deviation; On/Off, indicate times for contact (on) and dissociation phases (off) of each run. All peptides are C-terminally amidated. Peptides notated with * were designed and synthesised by Horsfall 2021 [1]. Changes to p21µ scaffold are indicated in bold.

Name	Sequence	ε_{205}*	Top Conc (nM)	Affinity K_D (nM)	K_D SD (nM)	On/Off (s)
p21 (139-160)	^{139}GRKRRQTSMTDFYHSKRRLIFS	98620	500	69.71	20.21	40/30
*p21µ	KRRQTSMTDFYHSKR	67860	1200	265.1	5.97	40/30
*p21µ-RD2	KRRQT**RI**T**EYF**HSKR	67380	150	20.33	6.84	40/30
*p21µ-Q144M	KRR**M**TSMTDFYHSKR	69290	32,000	41,400	820	40/30
*p21µ-T145K	KRRQ**K**SMTDFYHSKR	67860	128,000	10,000	65	40/30
*p21µ-T145D	KRRQ**D**SMTDFYHSKR	67860	64,000	4110	310	40/30
*p21µ-S146R	KRRQT**R**MTDFYHSKR	69210	1000	64.4	18.4	40/30
*p21µ-M147L	KRRQTS**L**TDFYHSKR	66030	5171.2	382	51.04	40/30
*p21µ-M147I	KRRQTS**I**TDFYHSKR	66030	500	37.05	7.84	40/30
*p21µ-D149E	KRRQTSMT**E**FYHSKR	67860	1000	400.66	45.54	40/30
*p21µ-F150Y	KRRQTSMTD**Y**YHSKR	65340	646.4	75.20	18.92	40/30
*p21µ-Y151F	KRRQTSMTDF**F**HSKR	70380	1292.8	167.2	19.07	40/30
*p21µ-FY150151YF	KRRQTSMTD**YF**HSKR	67860	1292.8	96.4	19.6	40/30
p21µ-AfumDNALIG	KRR**QRVRSIASFF**HSKR	74030	8000	458	117.77	40/30
p21µ-AfumDNAPOL	KRR**QKELSRFDF**HSKR	69900	10,000	659.3	105.8	40/30
p21µ-AfumFEN1	KRR**QSRLEGFF**HSKR	67120	10,000	713	56.9	40/30
p21µ-AfumRFC	KRR**MPTDIRNFF**HSKR	71730	2000	94.84	8.76	40/30

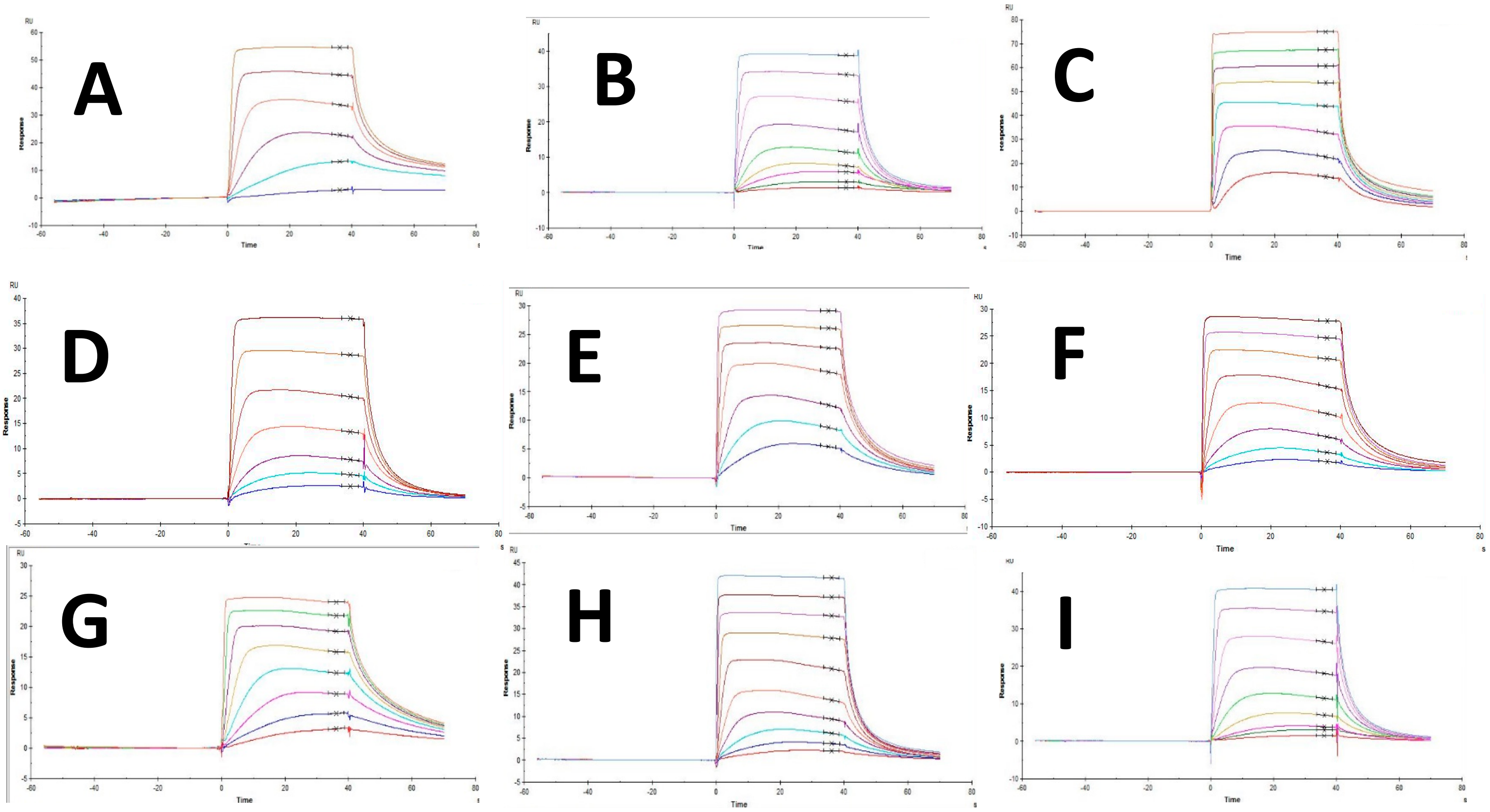

Figure S1. Representative sample of SPR sensorgrams testing peptide library against AfumPCNA. **A)** p21 (139-160). **B)** p21μ. **C)** p21μ-AfumRFC. **D)** p21μ-149E. **E)** p21μ-150Y. **F)** p21μ-151F. **G)** p21μ-147I. **H)** p21μ-147L. **I)** p21μ-RD2.

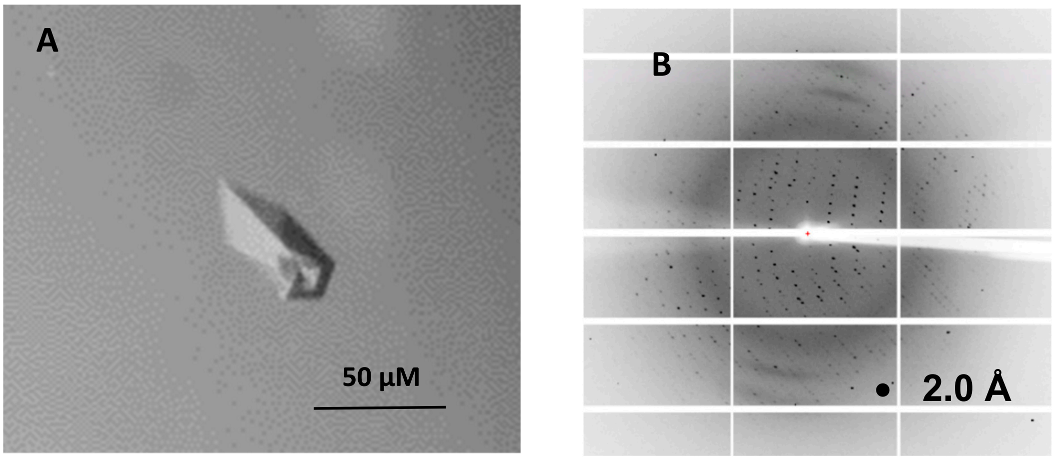

Figure S2. A) Protein crystal of AfumPCNA bound with p21μ crystal grown in 0.2M Tacsimate pH 4.0 0.1M Na Acetate, 16% PEG 3350 at 16 degrees Celsius after about 3 weeks. **B)** Diffraction image of protein crystal of AfumPCNA bound with p21μ diffracting at 2.0 Å.

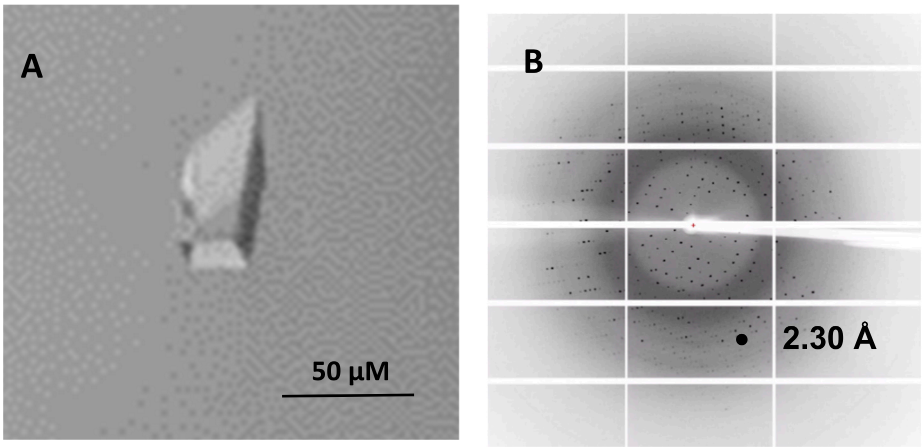

Figure S3. A) Protein crystal of AfumPCNA bound with p21µ-AfumRFC grown in 0.2M Tacsimate pH 4.0 0.1M Na Acetate, 16% PEG 3350 tray at 16 degrees celcius after 3 weeks. **B)** Diffraction image of protein crystal of AfumPCNA bound with p21µ-AfumRFC diffracting at 2.30 Å.

Table S2. Data collection and refinement statistics of AfumPCNA bound with p21µ structure (PDB:8GJF) and AfumPCNA bound with p21µ-AfumRFC structure (PDB:8GJ5). Statistics for the highest-resolution shell are shown in parentheses. Statistics for the highest-resolution shell are shown in parentheses.

**Parameter**	**8GJF**	**8GJ5**
Wavelength	0.9537	0.9537
Resolution range	36.55 - 2.0 (2.071 - 2.0)	47.2 - 2.3 (2.383 - 2.301)
Space group	C 1 2 1	P 21 21 21
Unit cell	146.0 84.8 70.4 90.0 91.4 90.0	83.9 97.0 108.1 90.0 90.0 90.0
Total reflections	114807 (11427)	79454 (7729)
Unique reflections	57621 (5742)	39748 (3880)
Multiplicity	2.0 (2.0)	2.0 (2.0)
Completeness (%)	99.1 (99.2)	99.8 (99.2)
Mean I/sigma(I)	13.2 (1.6)	21.0 (1.4)
Wilson B-factor	38.4	50.6
R-merge	0.032 (0.49)	0.024 (0.57)
R-meas	0.045 (0.69)	0.034 (0.81)
R-pim	0.031 (0.49)	0.024 (0.57)
CC1/2	0.99 (0.64)	1.00 (0.63)
CC*	1.00 (0.89)	1 (0.88)
Reflections used in refinement	57608 (5742)	39732 (3878)
Reflections used for R-free	2888 (263)	2021 (203)
R-work	0.23 (0.34)	0.23 (0.34)
R-free	0.27 (0.38)	0.25 (0.36)
CC(work)	0.95 (0.71)	0.94 (0.71)
CC(free)	0.92 (0.58)	0.94 (0.67)
Number of non-hydrogen atoms	6392	6018
macromolecules	5971	5874
solvent	421	144
Protein residues	803	797
RMS(bonds)	0.005	0.004
RMS(angles)	1.20	0.93
Ramachandran favored (%)	97.35	94.78
Ramachandran allowed (%)	2.28	4.59
Ramachandran outliers (%)	0.38	0.64
Rotamer outliers (%)	0.32	0.00
Clashscore	6.86	6.66
Average B-factor	50.00	58.67
macromolecules	49.85	58.75
solvent	52.20	55.44

[a] *Rmerge* = $\Sigma\ |I - \langle I \rangle| / \Sigma I$.

[b] *Rpim* = $\Sigma h\ [1/ ({}^{n}/_{h} - 1)]^{1/2}\ \Sigma i|\langle I_h \rangle - I_{h,i}| / \Sigma_h\ \Sigma_i\ I_{h,i}$ (2)

[c] *Rwork* = $\Sigma|F_o - F_c|/\Sigma|F_o|$ for all data excluding data used to calculate Rfree.

[d] *Rfree* = $\Sigma|F_o - F_c|/\Sigma|F_o|$ for all data.

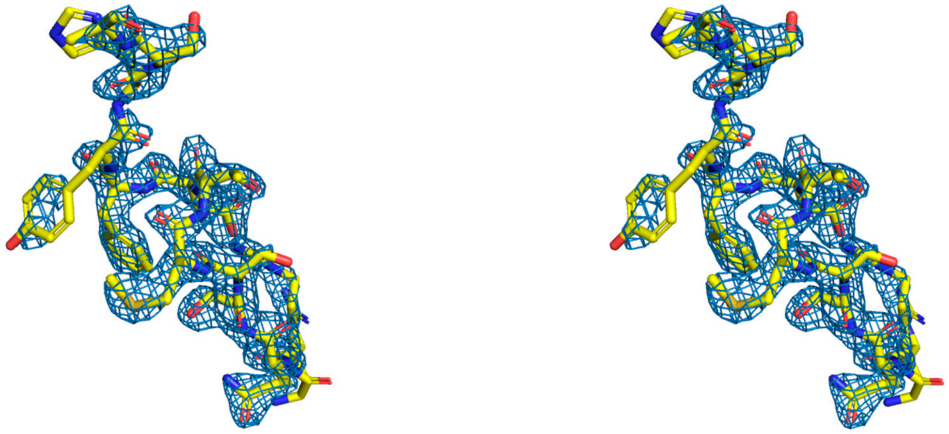

Figure S4. Representative electron density of **p21µ** (yellow, sticks) shown as a wall-eye stereo image $2F_o$-F_c composite omit map, view contoured at 1.5σ.

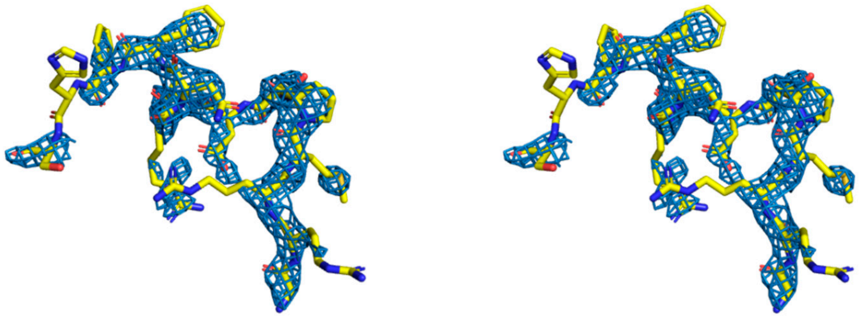

Figure S5. Representative electron density of **p21µ-AfumRFC** (yellow, sticks) shown as a wall-eye stereo image $2F_o$-F_c composite omit map, view contoured at 1.5σ.

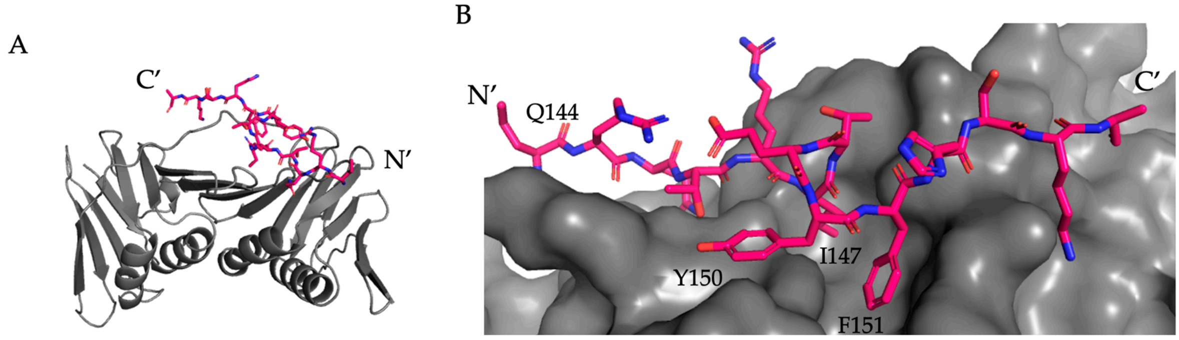

Figure S6. Computationally modelled structure of **p21μ-RD2** (pink, sticks) on the PIP-box binding site of a AfumPCNA monomer (grey, cartoon). A) Monomer. Peptide N and C terminus labelled. B) Surface, Conserved PIP box residues of peptide are labelled. Peptide N and C terminus labelled.

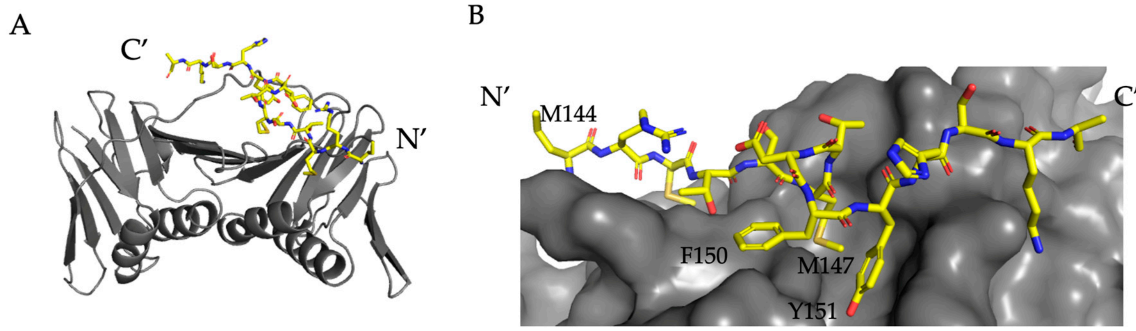

Figure S7. Computationally modelled structure of **p21$_{\mu}$-Q144M** (yellow, sticks) on the PIP-box binding site of a AfumPCNA monomer (grey, cartoon). A) Monomer. Peptide N and C terminus labelled. B) Surface, Conserved PIP box residues of peptide are labelled. Peptide N and C terminus labelled.

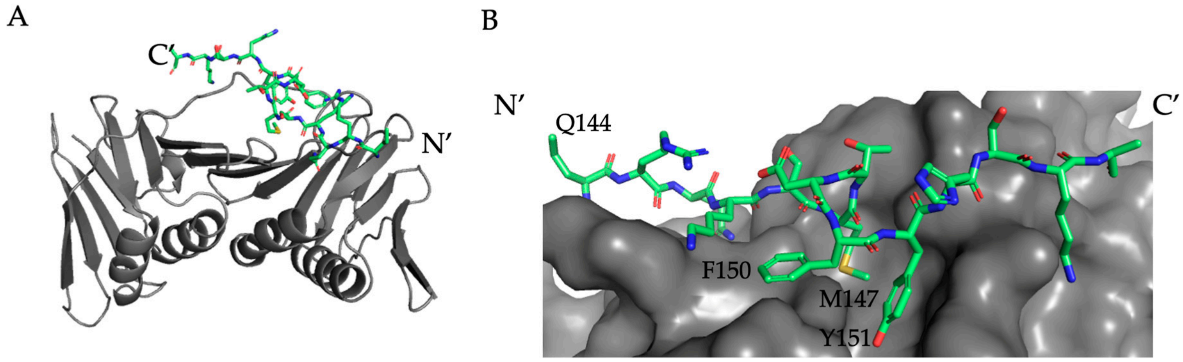

Figure S8. Computationally modelled structure of **p21$_{\mu}$-T145K (**lime green, sticks) on the PIP-box binding site of a AfumPCNA monomer (grey, cartoon). A) Monomer. Peptide N and C terminus labelled. B) Surface, Conserved PIP box residues of peptide are labelled. Peptide N- and C- terminus labelled.

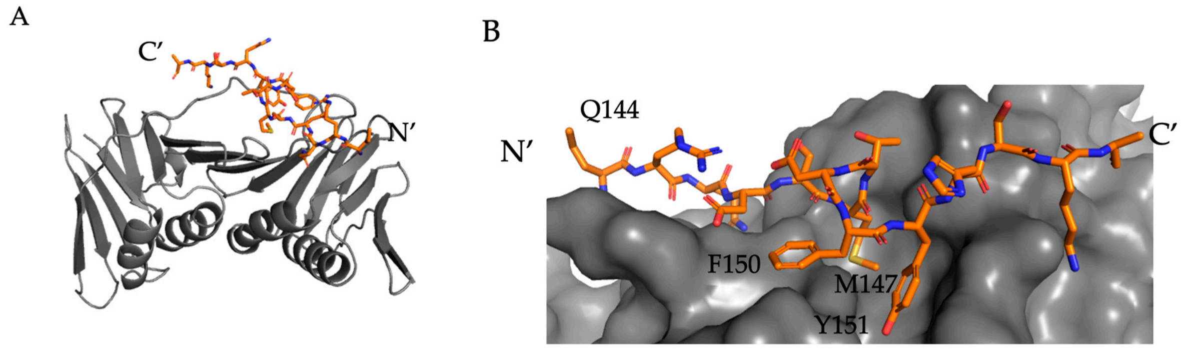

Figure S9. Computationally modelled structure of **$p21_{\mu}$-T145D** (orange, sticks) on the PIP-box binding site of a AfumPCNA monomer (grey, cartoon). A) Monomer. Peptide N and C terminus labelled. B) Surface, Conserved PIP box residues of peptide are labelled. Peptide N and C terminus labelled.

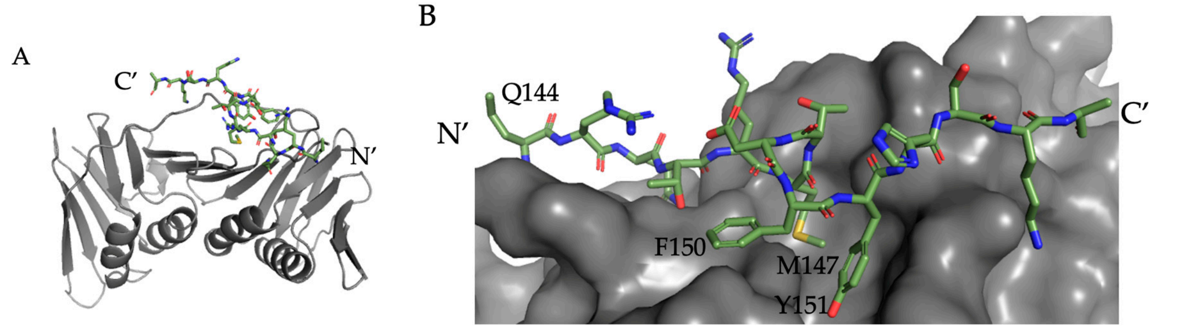

Figure S10. Computationally modelled structure of **$p21_{\mu}$-S146R** (olive, sticks) on the PIP-box binding site of a AfumPCNA monomer (grey, cartoon). A) Monomer. Peptide N- and C-terminus labelled. B) Surface, Conserved PIP box residues of peptide are labelled. Peptide N- and C-terminus labelled.

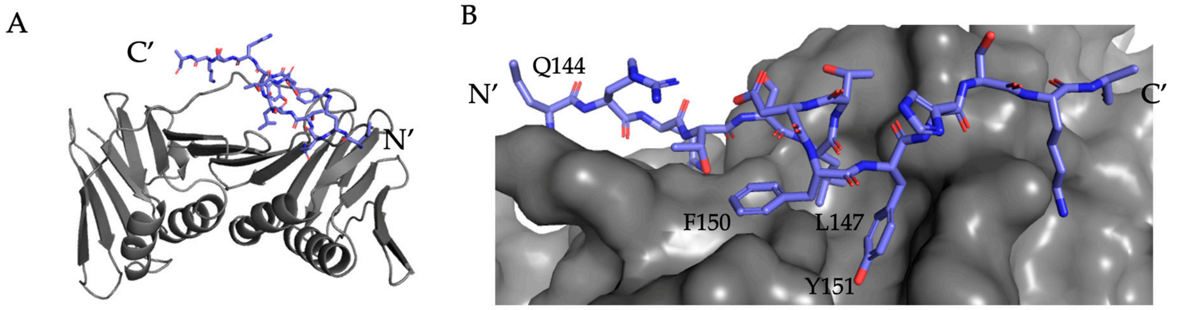

Figure S11. Computationally modelled structure of **$p21_{\mu}$-M147L** (lilac, sticks) on the PIP-box binding site of a AfumPCNA monomer (grey, cartoon). A) Monomer. Peptide N- and C-terminus labelled. B) Surface, Conserved PIP box residues of peptide are labelled. Peptide N- and C-terminus labelled.

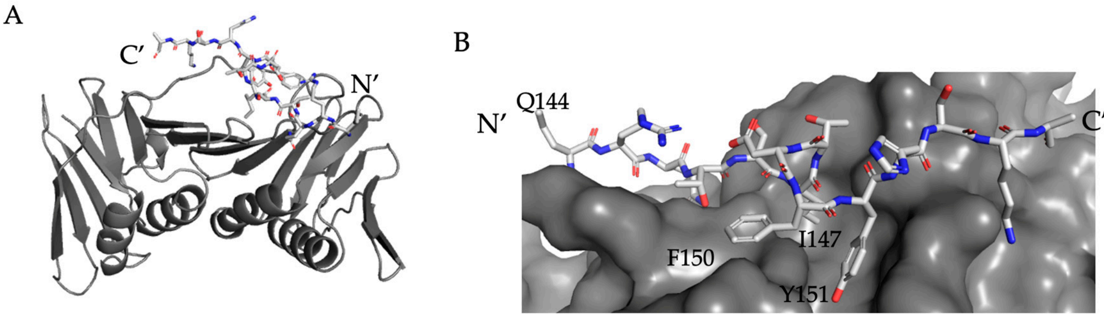

Figure S12. Computationally modelled structure of **$p21_{\mu}$-M147I** (light grey, sticks) on the PIP-box binding site of a AfumPCNA monomer (grey, cartoon). A) Monomer. Peptide N- and C-terminus labelled. B) Surface, Conserved PIP box residues of peptide are labelled. Peptide N- and C-terminus labelled.

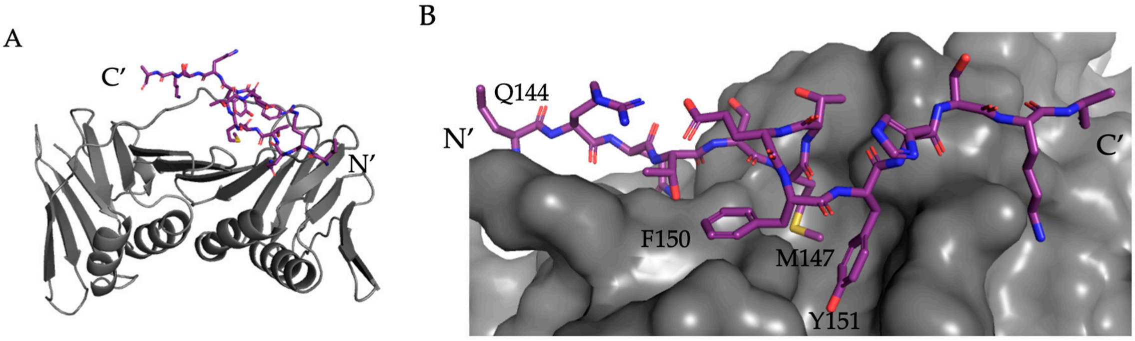

Figure S13. Computationally modelled structure of **$p21_{\mu}$-D149E** (purple, sticks) on the PIP-box binding site of a AfumPCNA monomer (grey, cartoon). A) Monomer. Peptide N- and C-terminus labelled. B) Surface, Conserved PIP box residues of peptide are labelled. Peptide N- and C-terminus labelled.

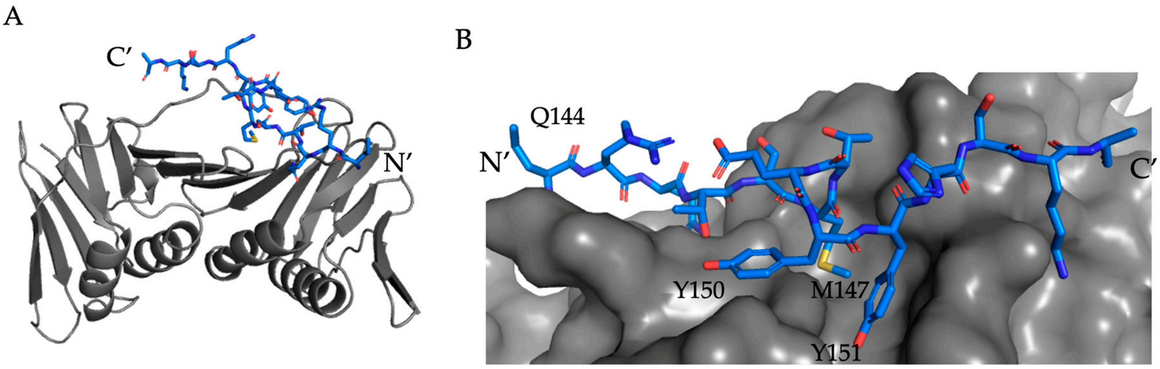

Figure S14. Computationally modelled structure of **p21μ-F150Y** (blue, sticks) on the PIP-box binding site of a AfumPCNA monomer (grey, cartoon). A) Monomer. Peptide N- and C-terminus labelled. B) Surface, Conserved PIP box residues of peptide are labelled. Peptide N- and C-terminus labelled.

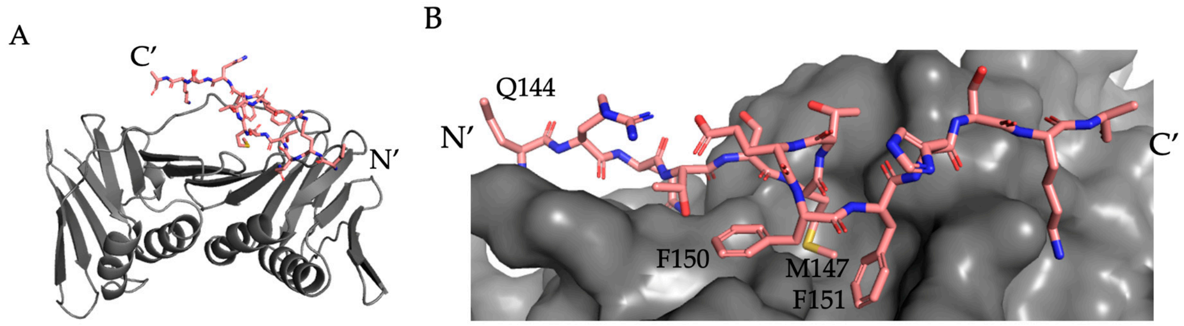

Figure S15. Computationally modelled structure of **p21µ-Y151F** (salmon, sticks) on the PIP-box binding site of a AfumPCNA monomer (grey, cartoon). A) Monomer. Peptide N- and C-terminus labelled. B) Surface, Conserved PIP box residues of peptide are labelled. Peptide N- and C-terminus labelled.

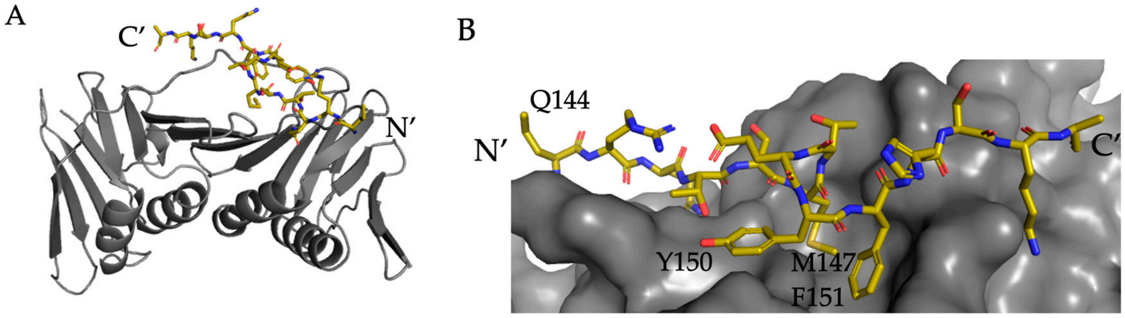

Figure S16. Computationally modelled structure of **p21µ-FY150151YF** (gold, sticks) on the PIP-box binding site of a AfumPCNA monomer (grey, cartoon). A) Monomer. Peptide N- and C-terminus labelled. B) Surface, Conserved PIP box residues of peptide are labelled. Peptide N- and C-terminus labelled.

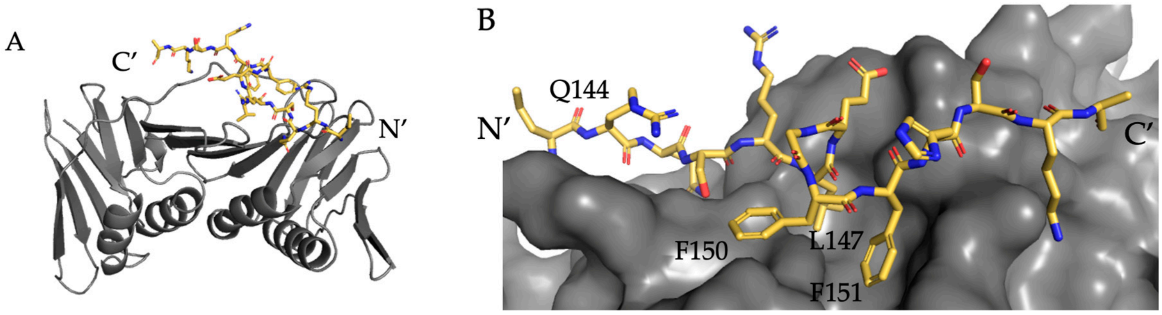

Figure S17. Computationally modelled structure of **p21µ-AfumFEN1** (yellow, sticks) on the PIP-box binding site of a AfumPCNA monomer (grey, cartoon). A) Monomer. Peptide N- and C-terminus labelled. B) Surface, Conserved PIP box residues of peptide are labelled. Peptide N- and C-terminus labelled.

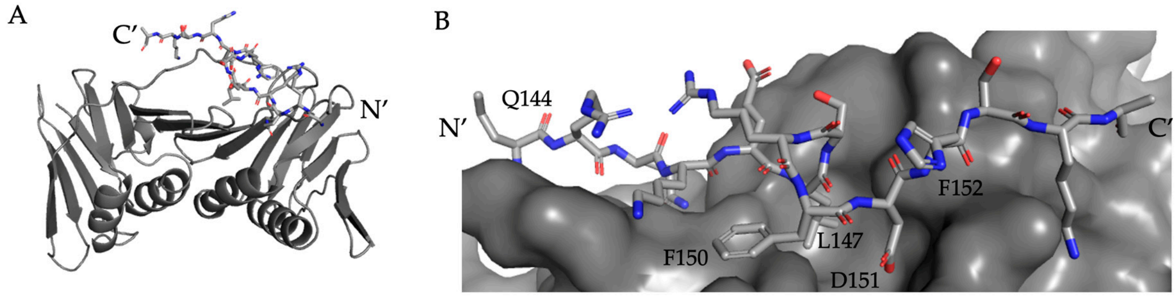

Figure S18. Computationally modelled structure of **p21µ-AfumDNAPOL** (grey, sticks) on the PIP-box binding site of a AfumPCNA monomer (grey, cartoon). A) Monomer. Peptide N- and C-terminus labelled. B) Surface, Conserved PIP box residues of peptide are labelled. Peptide N- and C-terminus labelled.

References

[1]Horsfall, A. J., Vandborg, B. A., Kowalczyk, W., Chav, T., Scanlon, D. B., Abell, A. D., and Bruning, J. B. Unlocking the PIP-box: A peptide library reveals interactions that drive high-affinity binding to human PCNA. *J Biol Chem* **(2021)** *296*, 100773, 10.1016/j.jbc.2021.100773.
